# Isoniazid preventive therapy in HIV-infected pregnant and postpartum women in high prevalence of tuberculosis countries: A protocol for systematic review

**DOI:** 10.1097/MD.0000000000023089

**Published:** 2020-11-20

**Authors:** Xiaozhuan Wang, Yun Zhang, Xiaojuan Lin, Yu Fu, Qingmei Sun, Jing Li, Xiaoling Liu, Jing Bai

**Affiliations:** Gansu Provincial Maternity and Child-care Hospital, Lanzhou, Gansu Province, China.

**Keywords:** HIV, isoniazid preventive therapy, pregnant and postpartum women, systematic review

## Abstract

**Background:**

Tuberculosis (TB) is the leading cause of health complications and death among human with immunodeficiency virus (HIV) infection. When TB develops during pregnancy or the early postpartum period, it is associated with negative maternal, pregnancy, and fetus and infant outcome, including premature birth, low birth weight, and congenital or neonatal TB infection or disease. The objective of this systematic review is to investigate the effective and safe of isoniazid for preventing TB for HIV-infected pregnant women in counties with high prevalence of TB.

**Methods:**

Pubmed, Embase, and Cochrane library will be searched to include randomized control trials which compared isoniazid preventive therapy with placebo for preventing TB in HIV-infected pregnant and postpartum women. RevMan version 5.3 will be used to perform all calculations related to the meta-analysis. Dichotomous data will be calculated in terms of a fixed or random effect model and expressed by the relative risk (RR) with 95% confidence interval (CI). The Cochrane collaboration's tool in the following aspects was used to assess the risk of bias (ROB) in included studies. The inconsistency index (I2) and Chi-squared will be applied for heterogeneity detection between clinical trials. A value of *P *< 0.05 will be considered statistically significant.

**Results:**

The main outcomes of pooled evidence synthesis will be presented including the incidence of TB and adverse events.

**Conclusion:**

This study will provide the evidence of whether isoniazid is an effective and safe intervention for preventing TB for HIV-infected pregnant women.

**Registration number:**

INPLASY202070011

## 1 Introduction

Tuberculosis (TB) is the leading cause of health complications and death among human with immunodeficiency virus (HIV) infection, especially in low-income and middle-income countries with a high tuberculosis burden.^[1]^ Tuberculosis predominantly affects those of reproductive age. Pregnant women with HIV are more likely to develop TB. It is reported that the risk for active tuberculosis disease is ranging from 0.7% to 7.9%, compared with 0.06% to 0.53% in HIV-uninfected women.^[2]^

When tuberculosis develops during pregnancy or the early postpartum period, it is associated with negative maternal, pregnancy, and fetus and infant outcome, including premature birth, low birth weight, and congenital or neonatal TB infection or disease.^[3–7]^ In South Africa, TB and pneumonia are the single most common cause of maternal mortality, accounting for more than 35% of all maternal deaths.^[8]^ Previous studies showed that pregnant women in HIV-infected with TB have 2-fold risk of delivering premature and low birth weight, and 6-fold in perinatal deaths.^[9]^

Effective preventive therapy of tuberculosis during pregnancy is essential for maternal and fetal health. Nowadays, the first-line treatment for TB consists of 4 drugs, isoniazid, rifampin, pyrazinamide, and ethambutol. However, relevant data are limited on the effect and efficacy of isoniazid preventive therapy in pregnant women with HIV-infected. It is recommended that initiation of isoniazid preventive therapy (IPT) in pregnant women with HIV by World Health Organization (WHO).^[10]^ However, several studies suggested that women who are pregnant or who have given birth in the previous 3 months have higher risk of liver injury.^[11,12]^ Therefore, this systematic review aimed to investigate the effective and safe of isoniazid for preventing TB for HIV-infected pregnant women in counties with high prevalence of TB.

## 2 Methods

This protocol was registered with the International Platform of Registered Systematic Review and Meta-Analysis Protocols (INPLASY, https://inplasy.com/) on July 03, 2020 and was last updated on July 03, 2020 (registration number INPLASY202070011, http://inplasy.com/inplasy-2020-7-0011/ ).^[13]^ Our review will develop following the Preferred Reporting Items for Systematic Reviews and Meta-Analyses (PRISMA) statement guidelines.^[14]^

### 2.1 Search strategy

**Figure 1:**
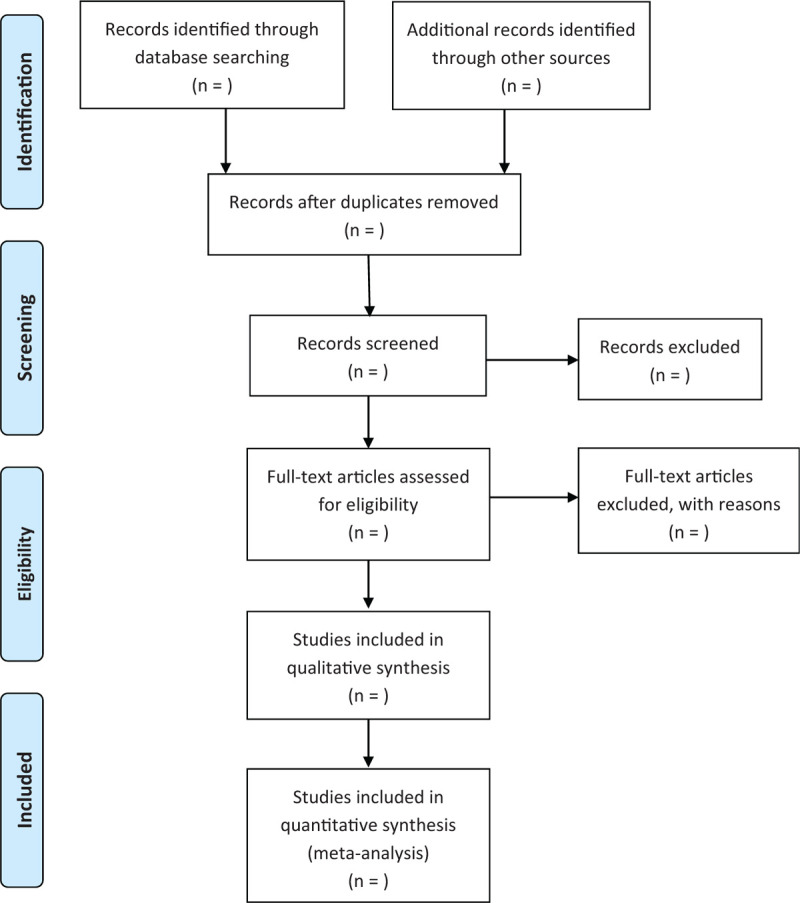
Flow diagram of study selection process.

Pubmed, Embase, and Cochrane library will be searched to include randomized control trials which compared isoniazid preventive therapy with placebo for preventing tuberculosis in HIV-infected pregnant and postpartum women. The search strategy will use keywords and mesh term including “isoniazid,” “human immunodeficiency virus,” “HIV,” and “pregnant women,” etc. Additional source including WHO clinical trial registry website, clinicaltrail.gov, conference abstracts, will also be searched. Further, the references of included trails will also be checked for more potential studies. The selection process will be presented in a PRISMA flow diagram (Fig. 1).

### 2.2 Eligibility criteria

This review will include trials assessing isoniazid preventive therapy versus placebo for preventing tuberculosis in HIV-infected pregnant and postpartum women in high prevalence of tuberculosis counties.

#### 2.2.1 Participants or population

Pregnant women, at 14 weeks through 34 weeks of gestation, who had HIV infection and were 18 years of age or older.

#### 2.2.2 Intervention

Isoniazid preventive therapy.

#### 2.2.3 Comparator

Placebo.

#### 2.2.4 Study designs

Only randomized controlled trials will be included.

#### 2.2.5 Main outcome(s)

The incidence of TB, adverse events including but not limited to hepatotoxicity and peripheral neuropathy.

### 2.3 Literature selection and data extraction

The retrieved records were imported into the EndNote X8 software and the duplicate publications were excluded. Two reviewers independently read the titles and abstracts of all identified records to exclude those that were clearly not relevant. Then the full texts of the articles retained were reviewed to further determine their suitability. Differences opinions were resolved by consensus.

The data were extracted by 2 reviewers independently using a predefined form. The following characteristics of included studies were collected: the first author, publication year, country, number of included patients, age, treatment duration, follow-up time, incidence of TB, adverse events, maternal, and infant outcomes. Any discrepancies were resolved by consensus.

### 2.4 Quality assessment

Two investigators will independently assess the risk of bias (ROB) in individual studies by using the Cochrane collaboration's tool in the following aspects: the assessment includes sequence generation; allocation concealment; blinding of participants, personnel, and outcome assessors; incomplete outcome data; selective outcome reporting; and other sources of bias. Any differences between the authors on the data extraction and quality assessment will be resolved by discussion.

### 2.5 Strategy of data synthesis

RevMan version 5.3 will be used to perform all calculations related to the meta-analysis. Dichotomous data will be calculated in terms of a fixed or random effect model and expressed by the relative risk with 95% confidence interval (CI). Continuous data will be presented as mean difference and 95% CI. The inconsistency index and *χ*^2^ will be applied for heterogeneity detection between clinical trials. When assessing the difference in outcome, heterogeneity involving all trials will be examined. A value of *P* < .05 will be considered statistically significant.

### 2.6 Subgroup analysis

When there is obvious heterogeneity among include studies, we will perform a subgroup analysis in accordance with different study qualities if possible.

### 2.7 Sensibility analysis

In the case of sufficient trials data, the ROB tool will be used to assess methodological quality. If low-quality articles are deleted, a second mete-analysis will be performed. The results and effect size of the 2 meta-analysis will be compared and discussed.

## 3 Discussion

TB is a global public health threat. There is a heavy disease burden, especially for the patients with HIV-infected in areas with limited resources. It is crucial that exploring effective and safety preventive approaches to decrease the incidence of TB among high-risk pregnant women. Moreover, during the pregnancy, physiologic changes occur that affect drug absorption, distribution, metabolism, and hepatic or renal clearance.^[15–17]^ There is no relevant systematic review that explored the effective preventive approach of IPT for pregnant women with HIV-infected. Additional Information section, URL of the online registry: https://inplasy.com/inplasy-2020-7-0011/

## Author contributions

**Data curation:** Xiaozhuan Wang, Yun Zhang.

**Methodology:** Xiaojuan Lin, Yu Fu.

**Project administration:** Sun QS, Li J

**Writing – original draft:** Xiaoling Liu.

**Writing – review & editing:** Jing Bai, Bai S.
